# Julia Woolnough Gross

**Published:** 1940

**Authors:** 


### Obituary

---

### JULIA WOOLNOUGH GROSS.

Miss Julia Gross died at the age of 74 at Ashburton, on 8th January. Her death recalls a long life spent in nursing at the Bristol Royal Infirmary, where she and her sister had worked for thirty-three years. Many generations of students and residents owed no small part of their training to the teaching of Sister Fanny and Sister Julia Gross. The two sisters were daughters of Mr. W. Gross, of Cheyne Square, Bury St. Edmund's.

They entered the nursing staff of the Bristol Royal Infirmary on the same day, 17th March, 1888, Fanny being aged 26 years and Julia 22. By September, 1892, Julia was made " Charge Nurse " of Wards 17 and 18, and she remained Sister of those wards until her retirement in 1921. In March, 1891, Fanny was put in charge of the operating theatre and was Theatre Sister until 1912, when she took charge of the Casualty Room.

They were women of unusual background and education for the times in which they took up nursing, and they were absolutely devoted to their profession. Professor James Swain writes :—

" Sister Julia Gross began her career in the days before the training of nurses had taken in the fuller methods of modern times. She came to the Royal Infirmary with her sister (' Sister Fanny,' who died a year ago) about 1885, when the clothes in which the patients arrived were kept in boxes under their respective beds and when there were only two baths for all the patients in the main building.

" In due course she attained the position of Sister in the men's surgical wards, and while others came and went, Sister Julia continued at her post at the Royal Infirmary until she sought a well-earned rest and retired, with her sister, to Ashburton, where they lived for the remainder of their lives in mutual happiness and content.

"Sister Julia was of a cheerful disposition, and her kindness and practical methods were appreciated and will be remembered by those who worked with her. Her death severs a link with the nursing world of the past which comparatively few can call to mind."

Dr. E. Mountjoy Pearse gives us his recollections of the two sisters :—

"Sister Fanny was always ready to help residents as well as dressers in any matter in her department. She taught the dressers all about surgical instruments and procedure at operations. In my memory she lives as a very kindly and efficient lady."

"Sister Julia," he writes, "had charge of Paul Bush's female wards. She was an excellent Sister to work with, a good disciplinarian, yet very popular with the nurses under her. She was always extremely well 'turned out,' 'every hair on duty,' so to speak. In my time all dressers acknowledged her as an expert on how to do or put on a dressing. No matter which surgeon they might be dressing for, every dresser used to endeavour to get Sister Julia to teach him how to put on a 'gum and chalk stocking,' for these in her hands looked like a plate from a book. Her wards were always in a state of being ready for inspection. She was indeed an excellent Ward Sister."

Their nephew, Flight-Lieut. E. C. Gross, R.A.F., M.S., who has kindly supplied information about their family, says :—

"Besides their wonderful devotion to their work the other wonderful thing was the love that each sister had for the other. They retired in 1921 to Brooklyn, Ashburton, where they spent the rest of their lives in a small cottage surrounded by reminders of a life-work well and cheerfully done."

